# Facile and Sustainable Synthesis of Commendamide and its Analogues

**DOI:** 10.3389/fchem.2022.858854

**Published:** 2022-03-01

**Authors:** Rosaria Villano, Francesco Tinto, Vincenzo Di Marzo

**Affiliations:** ^1^ Istituto di Chimica Biomolecolare, Pozzuoli, Italy; ^2^ Département de Médecine, Faculté de Médecine, Centre de Recherche de l’Institut Universitaire de Cardiologie et de Pneumologie de Québec, Université Laval, Quebec City, QC, Canada; ^3^ Canada Excellence Research Chair on the Microbiome-Endocannabinoidome Axis in Metabolic Health, Faculty of Medicine and Faculty of Agricultural and Food Sciences, Centre NUTRISS, Centre de Recherche de l’Institut de Cardiologie et Pneumologie de l’Université et Institut sur la Nutrition et les Aliments Fonctionnels, Université Laval, Quebec City, QC, Canada

**Keywords:** commendamide, microbiota, endocannabinoidome, green chemistry, organic synthesis, drug discovery

## Abstract

Commendamide, or N-(3-hydroxypalmitoyl)-glycine **1a**, is a gut microbiota-derived bioactive metabolite, structurally similar to long-chain N-acyl-amino acids which belong to the complex lipid signaling system known as endocannabinoidome and play important roles in mammals through activation of, *inter alia*, G-protein-coupled receptors (GPCRs). In this work, we describe a simple, green and economic method for the preparation of commendamide **1a**, a GPCR G2A/132 agonist. The developed protocol is general and could also be applied to the synthesis of deuterated commendamide **1b**, as well as to other minor microbiota-derived metabolites, such as the analog **2**.

## Introduction

The gut microbiota has proven to be an important source of bioactive metabolites (McNeil, 1984; De Vadder et al., 2014; Vernocchi et al., 2016; Postler and Ghosh, 2017), which are often able to interact with host receptors thanks to their structural similarity with endogenous signal molecules, such as those belonging to the endocannabinoidome (Iannotti and Di Marzo, 2021). This host signaling system includes hundreds of endocannabinoid-like long chain fatty acid-derived amides and esters, which signal at G-protein-coupled receptors (including the cannabinoid receptors), ligand-activated ion channels and peroxisome proliferator-activated receptors (PPARs) (Di Marzo, 2018). Gut microbiota metabolites can therefore act as highly specific modulators of important host functions and are very often well tolerated because biosynthesized by bacteria that live in symbiosis with the host. These aspects make bacterial endocannabinoidome-mimic molecules very promising candidates for the development of new drugs (Saha et al., 2016; Iannotti and Di Marzo, 2021).

In 2015, the discovery and structural identification of commendamide (Cohen et al., 2015) among the gut microbiota-derived metabolites aroused considerable interest due to its structure that is very similar to long-chain *N*-acyl-amino acids [such as *N*-oleoyl-glycine (Donvito et al., 2018)], which in the host signal through GPCRs (Cohen et al., 2017) and PPAR*α* (Donvito et al., 2018). In a very inspiring study (Cohen et al., 2015), Cohen et al. showed that commendamide was indeed able to interact with GPCRs by acting as an agonist of the GPCR G2A/132 receptor, implicated in autoimmunity and atherosclerosis. These interesting structural properties, its biogenic origin and bioactivity (Cohen et al., 2015; Lynch et al., 2017; Lynch et al., 2019; Piscotta et al., 2021), but also the presence of an *N*-(3-hydroxyacyl)amino acid scaffold, which is a structural motif found in many other interesting bioactive products (Morishita et al., 1997; Nemoto et al., 1998; Fuqua and Greenberg, 2002; Grandclément et al., 2016; Lynch et al., 2019), make of commendamide, a metabolite at the cross-road of gut microbiota and host signaling, an exceptionally attractive target for chemical synthesis.

Organic synthesis represents a powerful tool to conclusively confirm NMR and MS-based structure elucidation, and to produce greater amounts of the product, to be tested *in vitro* and *in vivo* (Nicolaou, 2014). Therefore, the elaboration of simple and effective synthetic protocols is very important; in addition, the development of versatile and general methodologies, which can also be used for the synthesis of the products in deuterated form (necessary for the development of LC-MS quantitative analysis methods) or to introduce structural modifications to the initial target molecule (for SAR studies) is highly desirable.

In the last decades, green chemistry (Horváth and Anastas, 2007; Li and Trost, 2008) has given impetus to a new generation of chemical syntheses through the creation of innovative reaction methodologies that can maximize the desired products and minimize waste, but also by identifying new synthetic sequences and equipments that can simplify the experimental procedures and replace old and often unsustainable protocols. These green syntheses, by employing more eco-friendly reaction conditions, represent a powerful tool in modern drug discovery programs.

**FIGURE 1:**
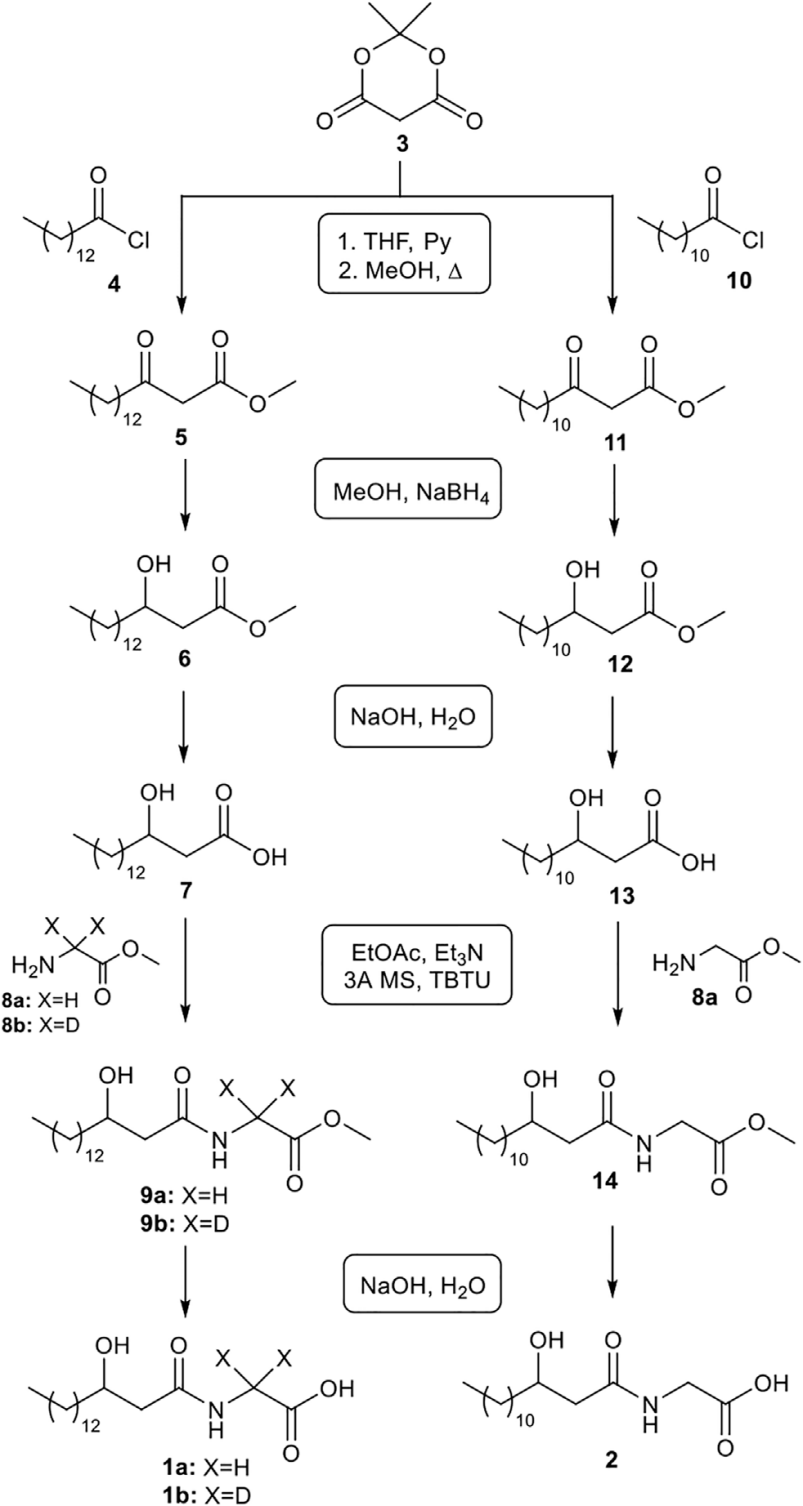
Syntheses of commendamide **1a**, deuterated commendamide **1b** and metabolite **2**.

Here, an easy and versatile methodology for the synthesis of the commendamide **1a** is reported. This synthetic sequence (Figure 1) is characterized by the use of simple workups, readily available reagents, no halogenated solvent (for reaction, workup and purification) and minimal volumes of organic solvents. Furthermore, the high yields observed in many reaction steps often rendered unnecessary the use of column chromatography for the purification of synthetic intermediates. The same synthetic sequence was also successfully applied to the synthesis of deuterated commendamide **1b** and another minor commendamide-like metabolite **2**, with a poorly studied bioactivity, thus demonstrating the generality of the novel synthetic route presented here.

## Methods

### General

All reagents and solvents were purchased from Merck-SigmaAldrich and used as received. Reactions were monitored by thin layer chromatography (TLC) on Merck silica gel plates (0.25 mm) and visualized by UV light at 254 nm and cerium sulfate reagent. ^1^H NMR and ^13^C NMR spectra were recorded on a Bruker Avance-400 and on a Bruker DRX 600 equipped with an inverse TCI CryoProbe at room temperature in CDCl_3_ or CD_3_OD. Chemical shifts are reported in ppm, multiplicities are indicated by s (singlet), d (doublet), t (triplet), q (quartet), m (multiplet) and br (broad). Coupling constants (J) are reported in Hz. Yields are given for isolated products showing one spot on a TLC plate and no impurities detectable in the NMR spectrum.

### Synthetic Procedures and Characterization of Products

*Methyl 3-oxohexadecanoate* (**5**): Pyridine (2 eq, 2.0 mmol) was added to a solution of Meldrum’s acid (**3**; 1 eq, 1.0 mmol) in THF (0.7 ml) at room-temperature. The reaction was cooled at 0°C and myristoyl chloride (**4**, 1.2 eq, 1.2 mmol) was portion-wise added to the solution. The reaction was warmed to rt and stirred overnight. After adding 1N HCl_aq_ to pH 2, the phases were separated and the H_2_O phase was extracted with EtOAc (3 × 2 ml). The combined organic layers were dried over Na_2_SO_4_. After evaporation of the solvent, the crude product was dissolved in MeOH (5 ml) and the reaction solution was mildly refluxed for 3 h by using a waterless air condenser (Asynt CondenSyn). After cooling to room temperature, the solvent was removed by reduced pressure and the crude product **5** was used directly without any further purification.

*Methyl 3-hydroxyhexadecanoate* (**6**): A solution of the product **5** in MeOH (1 ml) was prepared in a vial. The reaction was cooled at 0°C and NaBH_4_ (1 eq, 1.0 mmol) was added slowly; the reaction was stirred at 0°C for 30 min and, after adding 1N HCl_aq_ to pH 7, the reaction was warmed to rt. H_2_O (2 ml) and EtOAc (2 ml) were added to the reaction mixture, the phases were separated and the H_2_O phase was extracted with EtOAc (2 × 2 ml). The combined organic layers were dried over Na_2_SO_4_. After evaporation of the solvent, the crude product was purified by silica gel chromatography using a light petroleum ether/EtOAc 6/1 to give the product **6** (0.9 mmol, 90% yield from **3**). The spectroscopic data of **6** matched the ones reported in the literature (Jakob et al., 1996). ^1^H-NMR (CDCl_3_) *δ* 4.00 (m, 1H), 3.71 (s, 3H), 2.51 (dd, J = 3 Hz, 16.4 Hz, 1H), 2.40 (dd, J = 9.1 Hz, 16.4 Hz, 1H), 1.52-1.27 (m, 24H), 0.87 (t, J = 6.8 Hz, 3H). ^13^C-NMR (CDCl_3_) *δ* 173.5, 68.0, 51.7, 41.1, 36.5, 31.9, 29.7, 29.6, 29.5, 29.3, 25.5, 22.7, 14.1 (some signals were overlapped).

*3-Hydroxyhexadecanoic acid* (**7**): 1N NaOH_aq_ (10 eq, 2.5 mmol) was added to a solution of **6** (0.25 mmol) in THF (0.1 ml) at 0°C. The reaction was stirred at 0°C for 30 min and then 2 h at rt. After adding 1N HCl to pH 2, the phases were separated and the H_2_O phase was extracted with EtOAc (3 × 2 ml). The combined organic layers were dried over Na_2_SO_4_ and the solvent was removed by reduced pressure to afford *β*-hydroxy-acid **7** as a colorless solid (0.22 mmol, 88% yield). The spectroscopic data of **7** matched the ones reported in the literature (Bourboula et al., 2019). ^1^H-NMR (CDCl_3_) *δ* 4.02 (m, 1H), 2.58 (dd, J = 3.0, 16.6 Hz, 1H), 2.47 (dd, J = 9.0, 16.6 Hz, 1H), 1.57-1.25 (m, 24H), 0.88 (t, J = 6.8 Hz, 3H). ^13^C-NMR (CDCl_3_) *δ* 176.0, 68.0, 40.7, 36.5, 31.9, 29.7, 29.6, 29.57, 29.54, 29.5, 29.3, 25.4, 22.7, 14.1.

*Methyl* (*3-hydroxyhexadecanoyl*)*glycinate* (**9a**): The *β*-hydroxy-acid **7** (0.22 mmol) was dissolved in EtOAc (2 ml). 3 Å MS (400 mg), Et_3_N (3 eq, 0.66 mmol) and TBTU (1 eq, 0.22 mmol) were added and the reaction mixture was stirred at rt for 1 h. Then **8a** (Wang et al., 2009) (2.5 eq, 0.55 mmol) was added and the reaction was kept stirring overnight. The reaction mixture was diluted by adding 3 ml of H_2_O; the phases were separated and the H_2_O phase was extracted with EtOAc (3 × 2 ml). The collected organic layer was dried over Na_2_SO_4_ and concentrated. The residue was purified by flash chromatography using a light petroleum ether/ethyl acetate 7/3 to give **9a** (0.16 mmol, 73% yield). The spectroscopic data of **9a** matched the ones reported in the literature (Nemoto et al., 1998). ^1^H-NMR (CDCl_3_) *δ* 6.50 (bs, NH, 1H), 4.07 (dd, J = 5.4, 13.1 Hz, 1H), 4.03 (dd, J = 5.3, 13.1 Hz, 1H), 4.02 (m, 1H), 3.78 (s, 3H), 2.84 (bs, OH, 1H), 2.46 (dd, J = 2.5, 15.1 Hz, 1H), 2.35 (dd, J = 9.1, 15.1 Hz, 1H), 1.58-1.27 (m, 24H), 0.90 (t, J = 6.8 Hz, 3H). ^13^C-NMR (CDCl_3_) *δ* 172.9, 170.5, 68.8, 52.5, 42.7, 41.2, 36.8, 31.9, 29.7, 29.65, 29.58, 29.56, 29.5, 29.3, 25.5, 22.7, 14.1.

*Methyl* (*3-hydroxyhexadecanoyl*)*glycinate-d*_*2*_ (**9b**) was synthesized according to the same protocol as described for **9a**, by using **8b** (Wang et al., 2009) instead of **8a**, with 75% yield. ^1^H-NMR (CDCl_3_) *δ* 6.43 (bs, NH, 1H), 4.02 (m, 1H), 3.78 (s, 3H), 2.84 (bs, OH, 1H), 2.46 (dd, J = 2.5, 15.1 Hz, 1H), 2.34 (dd, J = 9.1, 15.1 Hz, 1H), 1.58-1.27 (m, 24H), 0.90 (t, J = 6.8 Hz, 3H). ^13^C-NMR (CDCl_3_) *δ* 172.8, 170.5, 68.7, 52.5, 42.7, 36.9, 31.9, 29.7, 29.66, 29.65, 29.58, 29.5, 29.3, 25.5, 22.7, 14.1.

*Commendamide* or *N-(3-hydroxypalmitoyl)-glycine* (**1a**): 1N NaOH_aq_ (10 eq, 1.3 mmol) was added to a solution of **9a** (0.13 mmol) in THF (0.1 ml) at 0°C. The reaction was stirred at 0°C for 30 min and then 3 h at rt. After adding 1N HCl to pH 2, the formation of a solid was observed. The solid product was collected by filtration and washed with H_2_O (2 × 1 ml) and then Etp/EtOAc 8/2 (3 × 1 ml) to give **1a** as a colourless solid (90% yield). Spectral data are consistent with a previous literature report (Cohen et al., 2015). ^1^H-NMR (CD_3_OD) *δ* 3.99 (m, 1H), 3.93 (ABq, J = 17.8 Hz, 2H), 2.41 (dd, J = 5.0, 14.3 Hz, 1H), 2.37 (dd, J = 7.7, 14.3 Hz, 1H), 1.52-1.31 (m, 24H), 0.92 (t, J = 6.8 Hz, 3H). ^13^C-NMR (CDCl_3_) *δ* 173.3, 171.7, 68.3, 43.2, 40.4, 36.7, 31.7, 29.4, 29.3, 29.2, 29.1, 25.2, 22.3, 13.0.

*Commendamide-d*_*2*_ or *N-*(*3-hydroxypalmitoyl*)*-glycine-d*_*2*_ (**1b**) was synthesized based on to the same protocol as described for **1a**, by using **9b** instead of **9a**, with 92% yield. ^1^H-NMR (CD_3_OD) *δ* 3.99 (m, 1H), 2.41 (dd, J = 5.0, 14.3 Hz, 1H), 2.37 (dd, J = 7.7, 14.3 Hz, 1H), 1.53-1.31 (m, 24H), 0.92 (t, J = 6.8 Hz, 3H). ^13^C-NMR (CDCl_3_) *δ* 173.3, 171.7, 68.3, 43.2, 36.7, 31.7, 29.4, 29.3, 29.2, 29.1, 25.2, 22.3, 13.0.

*Methyl 3-oxotetradecanoate* (**11**): Pyridine (2 eq, 2.0 mmol) was added to a solution of Meldrum’s acid (**3**; 1 eq, 1.0 mmol) in THF (0.7 ml) at room-temperature. The reaction was cooled at 0°C and lauroyl chloride (**10**, 1.2 eq, 1.2 mmol) was portion-wise added to the solution. The reaction was warmed to rt and stirred overnight. After adding 1N HCl_aq_ to pH 2, the phases were separated and the H_2_O phase was extracted with EtOAc (3 × 2 ml). The combined organic layers were dried over Na_2_SO_4_. After evaporation of the solvent, the crude product was dissolved in MeOH (5 ml) and the reaction solution was mildly refluxed for 3 h by using a waterless air condenser (Asynt CondenSyn). After cooling to room temperature, the solvent was removed by reduced pressure and the crude product **11** was used directly without any further purification.

*Methyl 3-hydroxytetradecanoate* (**12**): A solution of the crude product **11** in MeOH (1 ml) was prepared in a vial. The reaction was cooled at 0°C and NaBH_4_ (1 eq, 1.0 mmol) was added slowly; the reaction was stirred at 0°C for 30 min and, after adding 1N HCl_aq_ to pH 7, the reaction was warmed to rt. H_2_O (2 ml) and EtOAc (2 ml) were added to the reaction mixture, the phases were separated and the H_2_O phase was extracted with EtOAc (2 × 2 ml). The combined organic layers were dried over Na_2_SO_4_. After evaporation of the solvent, the crude was purified by silica gel chromatography using a light petroleum ether/EtOAc 6/1 to give the product **12** (0.75 mmol, 75% yield from **3**). The spectroscopic data of **12** matched the ones reported in the literature (Hon et al., 2007). ^1^H-NMR (CDCl_3_) *δ* 4.02 (m, 1H), 3.73 (s, 3H), 2.53 (dd, J = 3 Hz, 16.4 Hz, 1H), 2.43 (dd, J = 9.1 Hz, 16.4 Hz, 1H), 1.57-1.29 (m, 20H), 0.90 (t, J = 6.8 Hz, 3H). ^13^C-NMR (CDCl_3_) *δ* 173.5, 68.1, 51.7, 41.1, 36.5, 31.9, 29.7, 29.6, 29.5, 29.4, 29.3, 25.5, 22.7, 14.1.

*3-Hydroxytetradecanoic acid* (**13**): 1N NaOH_aq_ (10 eq, 7.5 mmol) was added to a solution of **12** (0.75 mmol) in THF (0.1 ml) at 0°C. The reaction was stirred at 0°C for 30 min and then 2 h at rt. After adding 1N HCl to pH 2, the phases were separated and the H_2_O phase was extracted with EtOAc (3 × 2 ml). The combined organic layers were dried over Na_2_SO_4_ and the solvent was removed by reduced pressure to afford *β*-hydroxy-acid **13** as a colorless solid (0.67 mmol, 89% yield). The spectroscopic data of **13** matched the ones reported in the literature (Fukuchi et al., 1992). ^1^H-NMR (CDCl_3_) *δ* 4.06 (m, 1H), 2.61 (dd, J = 3.1, 16.6 Hz, 1H), 2.51 (dd, J = 8.8, 16.6 Hz, 1H), 1.58-1.27 (m, 20H), 0.91 (t, J = 6.8 Hz, 3H). ^13^C-NMR (CDCl_3_) *δ* 176.0, 68.0, 40.7, 36.5, 31.9, 29.7, 29.6, 29.5 (some signals were overlapped), 29.3, 25.4, 22.7, 14.1.

*Methyl* (*3-hydroxytetradecanoyl*)*glycinate* (**14**): The *β*-hydroxy-acid **13** (0.67 mmol) was dissolved in EtOAc (6 ml). 3A MS (1 g), Et_3_N (3 eq, 2.0 mmol) and TBTU (1 eq, 0.67 mmol) were added and the reaction was stirred at rt for 1 h. Then **8a** (Wang et al., 2009) (2.5 eq, 1.7 mmol) was added and the reaction was kept stirring overnight. The reaction mixture was diluted by adding 6 ml of H_2_O; the phases were separated and the H_2_O phase was extracted with EtOAc (3 × 4 ml). The collected organic layer was dried over Na_2_SO_4_ and concentrated. The residue was purified by flash chromatography using a light petroleum ether/ethyl acetate 7/3 to give **14** (0.47 mmol, 70% yield). ^1^H-NMR (CDCl_3_) *δ* 6.40 (bs, NH, 1H), 4.11 (dd, J = 5.4, 18.3 Hz, 1H), 4.05 (dd, J = 5.4, 18.3 Hz, 1H), 4.02 (m, 1H), 3.79 (s, 3H), 2.88 (bs, OH, 1H), 2.46 (dd, J = 2.6, 15.1 Hz, 1H), 2.34 (dd, J = 9.1, 15.1 Hz, 1H), 1.58-1.27 (m, 20H), 0.90 (t, J = 6.5 Hz, 3H). ^13^C-NMR (CDCl_3_) *δ* 172.7, 170.5, 68.7, 52.5, 42.7, 41.1, 36.8, 31.9, 29.65, 29.63, 29.58, 29.5, 29.3, 25.5, 22.7, 14.1.

*3-Hydroxytetradecanoyl-glycine* (**2**): 1N NaOHaq (10 eq, 4.7 mmol) was added to a solution of **14** (0.47 mmol) in THF (0.1 ml) at 0°C. The reaction was stirred at 0°C for 30 min and then 3 h at rt. After adding 1N HCl to pH 2, the formation of a solid was observed. The solid product was collected by filtration and washed with H_2_O (2 × 2 ml) and then Etp/EtOAc 8/2 (3 × 2 ml) to give **2** as a colourless solid with 89% yield (0.42 mmol). This spectral data is consistent with a previous literature report (Morishita et al., 1997; Cohen et al., 2015). ^1^H-NMR (CD_3_OD) *δ* 3.99 (m, 1H), 3.94 (ABq, J = 17.8 Hz, 2H), 2.41 (dd, J = 5.0, 14.3 Hz, 1H), 2.37 (dd, J = 7.7, 14.3 Hz, 1H), 1.53-1.31 (m, 20H), 0.92 (t, J = 6.8 Hz, 3H). ^13^C-NMR (CDCl_3_) δ 173.3, 171.7, 68.3, 43.2, 40.3, 36.7, 31.7, 29.4, 29.35, 29.34, 29.3 (some signals were overlapped), 29.1, 25.2, 22.3, 13.0.

## Results and Discussion

As delineated in Figure 1, 3-hydroxypalmitic acid **7** was the key intermediate for the production of commendamide **1a**. Several methods for the synthesis of 3-hydroxy carboxylic acids are reported in the literature and they mainly use the Reformatsky reaction (Gawrorisk, 1984) or the reduction of *β*-ketoesters obtained by acylation of Meldrum’s acid (Oikawa et al., 1978; Hodgkinson et al., 2011; Brinkerhoff et al., 2014; Brosge et al., 2021) or *γ*-alkylation of acetoacetate (Smith et al., 2009). Here, we started from the reaction between the commercially available Meldrum’s acid **3** and myristoyl chloride **4**. Unlike the protocols reported in literature, this C-acylation was carried out in a minimal volume of THF (workup with EtOAc) instead of classical CH_2_Cl_2_, without any reduction in the efficiency of the reaction. Because the acyl Meldrum’s acid derivatives are decomposed by column chromatography, the crude product was used without any further purification and directly converted to methyl 3-oxohexadecanoate **5** by alcoholysis with methanol. This reaction was performed at 72–75°C, by classical heating (3 h) in the presence of a waterless air condenser (Asynt CondenSyn)^1^, an eco-friendly equipment that reduces water waste. The crude *β*-ketoester **5** was swiftly converted into *β*-hydroxy-ester **6** by reduction with NaBH_4_ in MeOH (30min/0°C) and the product **6** was easily isolated by column chromatography (90% yield from **3**, after 3 reaction steps). Saponification of the methyl ester group with NaOH in water gave the corresponding *β*-hydroxy-acid **7** as a colorless solid in 88% yield. In order to increase the solubility of product **6** and improve the efficiency of the reaction, a small volume (100 μL) of THF was also added as co-solvent. TBTU-mediated coupling of **7** with glycine methyl ester hydrochloride **8a** [prepared according to the literature (Wang et al., 2009)], in the presence of Et_3_N and 3 Å MS provided the glycinate **9a** with an interesting yield (73%) after purification by flash chromatography. Finally, commendamide **1a** was obtained as a colourless solid by saponification of the derivative **9a** and, after crystallization, was collected by filtration (total yield 52% from **3**).

The synthesis of commendamide was reported by Cohen in 2015 (Cohen et al., 2015). In this case the racemic product was prepared by a coupling reaction between 3-hydroxy-palmitic acid and glycine promoted by PyBOP and Cl-HOBt. Unfortunately, the article does not provide sufficient synthetic details and the final product was obtained in very small amount (less than 6% of yield).

The synthetic sequence from **3** to **1a** was rather concise (6 steps) and all reactions gave the corresponding products with good yields, without formation of side-products. All reactions of this sequence were not very water-sensitive, so they did not need to be carried out under an inert atmosphere, nor did they require the use of anhydrous reagents, solvents, glassware, and equipments. Furthermore, most of the reactions were realized at room temperature, while low temperatures (0°C) and high temperatures (75°C) were used only in a few steps for very limited times. In addition, all workups were very simple and column chromatography was performed only for the purification of two synthetic intermediates, since the other products were used in crude form or after purification by crystallization and filtration, with a significant and beneficial reduction in the amount of solvent and energy needed for the separation and purification of intermediates. Finally, this synthetic sequence was readily scalable (up to 5 mmol) without substantial change in efficiency.

The same synthetic protocol was also applied to the synthesis of deuterated commendamide **1b** (Figure 1); in this case, deuterated glycine methyl ester hydrochloride **8b** [prepared according to the literature (Wang et al., 2009)], was used in the coupling reaction with 3-hydroxypalmitic acid **7**. Deuterated commendamide **1b** was synthesized with a total yield of 55% from **3**.

To further explore the synthetic value of this strategy, we extended it to the synthesis of another poorly studied gut-microbiota metabolite, that is the commendamide analog **2** (Figure 1). In this case, C-acylation of the Meldrum’s acid **3** was performed with lauroyl chloride **10**, then the alcoholysis with MeOH, followed by reduction with NaBH_4_ and saponification gave *β*-hydroxy-myristic acid **13**. Finally, TBTU-mediated coupling of **13** with glycine methyl ester hydrochloride **8a** followed by saponification produced the derivative **2** with 42% overall yield from **3**, which is higher than that reported in the literature by using a different synthetic protocol (Venkateswaran et al., 2016).

**FIGURE 2:**
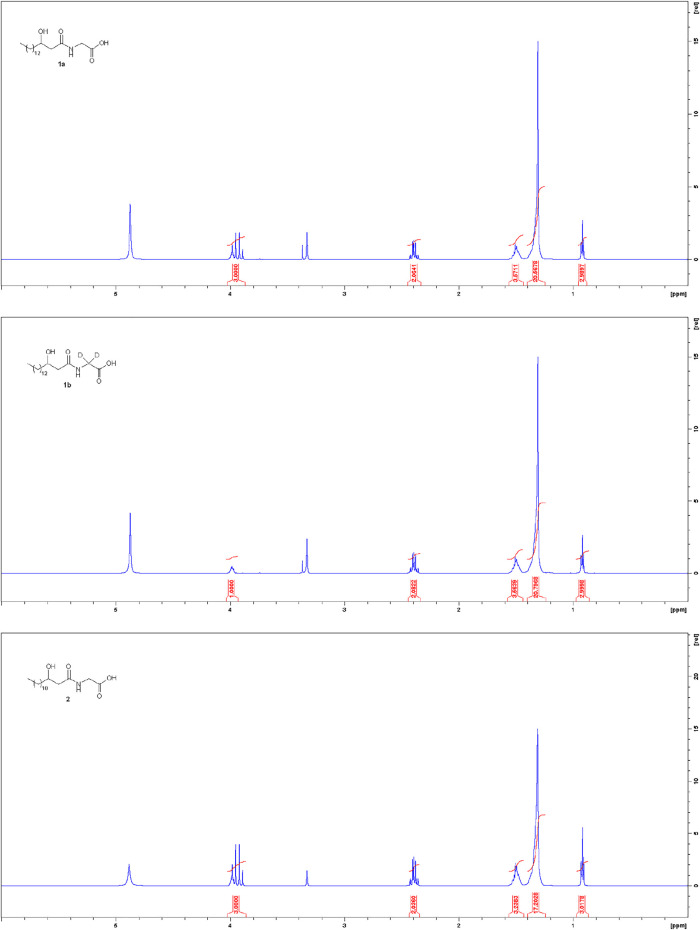
^1^H NMR spectra (600MHz, CD_3_OD) of synthetic products **1a**, **1b** and **2**.

The possibility to apply the same synthetic sequence for the production of several products with a 3-hydroxyacyl glycine scaffold (highly pure **1a**, **1b** and **2**, Figure 2) confirmed the generality and versatility of the developed strategy.

## Conclusion

In conclusion, a practical and “green” procedure for the synthesis of commendamide and its analogues was developed. This procedure involved a total of six steps to obtain highly pure N-(3-hydroxyacyl) glycines (Figure 2) from the commercially available Meldrum’s acid and acyl-chloride. Key advantages are that all reactions of this synthetic sequence gave the corresponding products with high yields, without formation of side-products, and only two column chromatographic purifications were needed with a beneficial reduction in the amount of solvent and energy needed for separation and purification; furthermore, all reactions were not very water-sensitive, so they did not need to be carried out under an inert atmosphere, and they did not require the use of anhydrous reagents/solvents/glassware.

The possibility to apply the same synthetic sequence for the production of several products with a 3-hydroxyacyl glycine scaffold (**1a**, **1b** and **2**) confirmed the generality and versatility of the developed strategy. Given the simplicity and high efficiency of this synthetic sequence, the extension of this methodology toward the synthesis of other gut microbiota-derived commendamide-like metabolites (Cohen et al., 2017) for biological evaluation, *in vitro* and *in vivo*, are underway in our laboratory. Finally, the separation of the single enantiomers by chiral HPLC will allow to test their individual biological activities and evaluate any differences between the stereoisomers (in addition to the biological tests performed on the racemic products).

## Data Availability

The original contributions presented in the study are included in the article, further inquiries can be directed to the corresponding author.

## Footnotes

1
http://www.asynt.com/product/asyntcondensyn-air-condenser/
